# SARS-CoV-2 Infection in Dogs and Cats: Facts and Speculations

**DOI:** 10.3389/fvets.2021.619207

**Published:** 2021-02-10

**Authors:** Nicola Decaro, Andrea Balboni, Luigi Bertolotti, Piera Anna Martino, Maurizio Mazzei, Francesco Mira, Ugo Pagnini

**Affiliations:** ^1^Department of Veterinary Medicine, University of Bari Aldo Moro, Bari, Italy; ^2^Department of Veterinary Medical Sciences, Alma Mater Studiorum - University of Bologna, Bologna, Italy; ^3^Department of Veterinary Science, University of Torino, Turin, Italy; ^4^Department of Veterinary Medicine, University of Milano, Milan, Italy; ^5^Department of Veterinary Sciences, University of Pisa, Pisa, Italy; ^6^Istituto Zooprofilattico Sperimentale della Sicilia “A. Mirri”, Palermo, Italy; ^7^Department of Veterinary Medicine and Animal Productions, University of Naples “Federico II”, Naples, Italy

**Keywords:** SARS-CoV-2, cats, dogs, facts, speculations

## Introduction

The recent pandemic caused by the novel human coronavirus (CoV), currently referred to as severe acute respiratory syndrome coronavirus type 2 (SARS-CoV-2), which is responsible for COronaVIrus Disease 2019 (COVID-19), is leading to alarmism among pet owners as a consequence of few case reports of SARS-CoV-2 infection in dogs and cats. COVID-19 emerged in December 2019 in Wuhan City, Hubei Province, China, in humans exposed to wildlife at the Huanan seafood wholesale market, the largest seafood market in central China. This is a typical Asian wet market, where different species of farm and wild animals are commonly sold dead and live. The blood and other body fluids originating from these animals represent an exceptional source for the spillover of animal viruses (1).

SARS-CoV-2 recognizes a probable zoonotic origin, since the virus likely descends from a bat betacoronavirus, strictly related to the one responsible for the 2002–2003 SARS epidemic (SARS-CoV), which was transmitted to humans directly or through previous adaptation to a not yet identified intermediate host (1, 2). After this adaptation to the new host, the virus was able to spread to the human population through a human-to-human transmission, without any further role of animals in the epidemiological chain. However, pets have been alternatively brought into play as a possible source of infection for humans, intermediate hosts for SARS-CoV-2 transmission to humans or hosts of animal CoVs that may cross-protect humans against the highly pathogenic CoV. The aim of this opinion article is to define the role of dogs and cats in the SARS-CoV-2 epidemiology in the light of current knowledge.

## Can Dogs and Cats be Infected by SARS-CoV-2?

To date, sporadic cases of SARS-CoV-2 infection have been reported in dogs and cats. The first animal cases involved 2 dogs, a 17-year-old Pomeranian dog and a 2-year-old German shepherd, living in Hong Kong and in close contact with SARS-CoV-2 infected human patients (3). The animals did not display any clinical signs related to the infection and they shed, in their respiratory secretions, low SARS-CoV-2 loads as measured by real-time RT-PCR, a molecular tool able to detect even traces of viral RNA. Accordingly, a mixed breed dog living with the infected German shepherd (that also developed antibodies against SARS-CoV-2) has remained uninfected. Unfortunately, the Pomeranian dog died a few days after repeatedly testing negative for SARS-CoV-2 and the owner declined any necropsy, but the cause of death was clearly associated to the previous heart and kidney disease affecting this old animal. A third SARS-CoV-2 positive dog was later reported in North Carolina, USA. The infected pug displayed mild respiratory signs (sneeze and cough) and was living in a highly contaminated household, where 3 family members tested positive for SARS-CoV-2. The family participated in a study at Duke University that involved testing family members and their pets for COVID-19^1^. However, the United States Department of Agriculture's National Veterinary Services Laboratories were unable to confirm the positive testing of this pug. The first confirmed case of SARS-CoV-2 infection in a pet dog in the United States was then reported in a German shepherd in the State of New York^2^.

A greater concern was, on the other hand, generated by the involvement of cats. The first case occurred in a cat in Brussels whose owner had just returned from a vacation in Italy (4). The cat developed a gastroenteric disease. It shed discrete SARS-CoV-2 titres in the vomit and, to a lesser extent in the feces. To date, whether the observed clinical signs were associated with SARS-CoV-2 infection is unknown. Another SARS-CoV-2 infected cat was reported in Hong Kong. The pet was living with an infected human patient and did not display any clinical signs, but the virus was detected in its respiratory secretions and feces^3^. Two cats living in two separate areas of the State of New York and having mild respiratory illness tested SARS-CoV-2 positive. The first cat was tested after it showed mild respiratory signs, but no individuals in the household were confirmed to be ill with COVID-19. The source of infection of the cat might have been the contact with an infected human inside or outside its household. The second cat showed signs of respiratory illness after its owner tested positive for COVID-19. Both animals fully recovered from the respiratory disease (5). Sporadic cases of SARS-CoV-2 natural infection in dogs and cats were reported throughout the world (6). A high prevalence of PCR positive testing was reported in cats in Hong Kong, with 6 cases (out of 50 quarantined animals from COVID-19 positive households) of apparent human-to-feline transmission involving healthy cats (7).

To date, despite these few reports of SARS-CoV-2 infections in pets, there is some evidence that cats may be more susceptible than dogs to this highly pathogenic human CoV. SARS-CoV-2 is strictly related to SARS-CoV at genetic and biological levels, being included in the same viral species, *Severe acute respiratory syndrome-related coronavirus* (subgenus *Sarbecovirus*, genus *Betacoronavirus*) and sharing the same cellular receptor, the angiotensin converting enzyme type 2 (ACE2) (8). SARS-CoV-2 infected cats both through the natural and the experimental routes. Several cats of the Amoy Gardens in Hong Kong, where more than 100 infected people were living, were found to be positive for SARS-CoV^4^ and the experimental administration of this virus resulted in a productive infection with shedding of high viral loads and virus transmission to in-contact cats (9). *In-silico* analysis of the feline and ferret ACE2 has shown that SARS-CoV-2 can bind with high efficiency to the receptors of these animals (10). Dogs experimentally inoculated with SARS-CoV-2 developed a mild infection and shed low titres of viral RNA. In contrast, experimentally-infected cats and ferrets appeared to be highly susceptible to SARS-CoV-2, shedding high amounts of virus and infecting (few) in-contact animals (11). A serological survey was conducted in cats in Wuhan (China), the epicenter of the CoV pandemic, between January and March 2020. Fifteen out of 102 cats tested positive by an ELISA using the receptor-binding domain (RBD) of the spike protein, and 11 of these positivities were confirmed by virus neutralization. Most of the seropositive animals had been in close contact with SARS-CoV-2 infected humans (12). In a large-scale study assessing SARS-CoV-2 infection in 919 companion animals living in northern Italy, sampled at a time of frequent human infection, none of these pets tested PCR positive. However, 3.3% of dogs and 5.8% of cats had measurable SARS-CoV-2 neutralizing antibody titres; dogs from COVID-19 positive households were significantly more likely to test positive than those from COVID-19 negative households (13). Higher seroprevalence rates for SARS-CoV-2, ranging from 21 to 53% depending of the test used, were observed in domestic carnivores from COVID-19 positive household in two French regions (14). On the contrary, no SARS-CoV-2 antibodies nor viral RNA were detected in 21 domestic pets (9 cats and 12 dogs) living in close contact with a veterinary community with some COVID-19 positive students (15).

## Are Dogs Involved in SARS-CoV-2 Emergence?

A recent paper has suggested the dog as an intermediate host for SARS-CoV-2 adaptation and transmission to humans (16). The hypothesis is based on the low frequency of CG dinucleotides (CpG) in the SARS-CoV-2 genome, which was much lower with respect to other betacoronaviruses, but similar to that observed in the canine alphacoronavirus (CCoV), with particular regards to that of a hypervirulent strain (pantropic CCoV) first detected in Italy (17, 18), and later reported in other countries (19, 20). The reduced number of CpGs may represent a pathogenicity marker, since these dinucleotides are the target for a cellular antiviral protein, the zinc finger antiviral protein (ZAP). ZAP binds specifically to CpG dinucleotides in viral RNA genomes via its RNA-binding domain, thus inhibiting viral replication and mediating viral genome degradation. As a consequence, a lower CpG content in the viral genome is associated with a greater ZAP resistance and higher virulence. A further hypothesis of the same study is that SARS-CoV-2 was originally an enteric virus, since the enteric environment seems to be more favorable to the selection of viruses with low CpG content and indeed CCoV, the canine low CpG content alphacoronavirus, replicates in the gut mucosa (21). Accordingly, a number of human patients with COVID-19 display intestinal symptoms (22). Certainly, the possible role of dogs as intermediate hosts is a fascinating hypothesis, but at the moment it appears just a speculation lacking any robust scientific evidence. A snake origin of the virus was suggested on the basis of the codon usage bias of SARS-CoV-2 that seemed to be more suitable for the transcription machinery of reptiles (23). However, this hypothesis was subsequently rebutted due to the lack of robust data (8).

## May Feline and Canine Coronaviruses Cross-Protect Humans Against SARS-CoV-2 Infection?

Dogs and cats have their own CoVs, which are genetically and biologically divergent from SARS-CoV-2 and do not infect humans (8). Two CoVs are currently circulating in dogs. The enteric alphacoronavirus CCoV forms a unique viral species along with transmissible gastroenteritis virus of swine (TGEV) and feline coronavirus (FCoV). The betacoronavirus canine respiratory coronavirus (CRCoV) is closely related to bovine coronavirus (BCoV) and to a low-pathogenic betacoronavirus of humans, HCoV-OC43 (8). CCoV includes two genotypes, CCoV-I and II. The latter consists of two subgenotypes, CCoV-IIa and IIb, which are represented by classical and recombinant TGEV-like strains, respectively (24, 25). Hypervirulent strains belong to subtype CCoV-IIa (6). In cats two genotypes (FCoV-I and FCoV-II) are known, both including two different pathotypes, namely the feline enteric coronavirus and the feline infectious peritonitis virus. The former is responsible for asymptomatic infections or mild, self-limiting enteritis while the latter causes a systemic, fatal disease known as feline infectious peritonitis (8). An Italian study, based on computational analysis of full-length CoV genomes, has recently identified some immunorelevant epitopes in the RBD of the spike protein, which seem to be conserved between SARS-CoV-2 and some animal betacoronaviruses, mainly BCoV and CRCoV (26). According to the authors' hypothesis, repeated exposure to these animal CoVs may have partially immunized people against SARS-CoV-2. This hypothesis is fascinating but needs to be further investigated. A recent study has demonstrated that humans previously exposed to other antigenically distinct common seasonal HCoVs could have non-neutralizing antibodies that cross-reacted with SARS-CoV-2. These antibodies were not associated with protection against SARS-CoV-2 infections or hospitalizations, but paradoxically they were boosted upon SARS-CoV-2 infection (27).

However, the presence of poorly cross-reacting antibodies may instead trigger an antibody-dependent enhancement favoring the entry into the host cells of viral particles bound to cytophilic antibodies (28). Similar protection against or exacerbation of COVID-19 should have been elicited by HCoV-OC43, a low pathogenic betacoronavirus that has been circulating in humans for more than a century (8). Therefore, in this case also, stronger scientific evidence is needed to confirm the hypothesis suggested on the basis of a raw bioinformatics analysis.

## Discussion

To date (December 14, 2020), no pet-to-human transmission of the virus has been reported during this phase of the pandemic and although more than 71 million humans in the world tested positive for SARS-CoV-2 (https://www.ecdc.europa.eu/en/geographical-distribution-2019-ncov-cases), there are only sporadic cases of natural infections known in dogs and cats. Therefore, according to the present knowledge, the fact that SARS-CoV-2 can sporadically infect some domestic carnivores does not imply that pets play an active role in the virus transmission to humans. However, there is increasing evidence that, albeit with different ranges of susceptibility, dogs and cats can be infected as a consequence of close contact with SARS-CoV-2 positive people. In this scenario, dogs and cats act as (often asymptomatic) victims of a human-to-pet transmission rather than the source of infection for human beings.

In addition, studies that rely only on bioinformatic and computational analyses of viral genomes, mRNAs and proteins may outline interesting scenarios about SARS-CoV origin, epidemiology and pathobiology, but they need to be supported by more comprehensive *in-vitro* and *in-vivo* investigations. Accordingly, taking into account the scientific evidence supporting the current knowledge, dogs and cats are unlikely to play any role in the emergence and/or transmission to humans of this pandemic virus. Only extensive epidemiological investigations in dogs and cats living in geographic areas with high prevalence of SARS-CoV-2 in humans can definitively clarify their role in the transmission of the virus. In addition, more comprehensive seroepidemiological studies in veterinarians, pet owners and breeders should assess whether these population groups are either (partially) protected from COVID-19 or susceptible to a more severe course of SARS-CoV-2 infection.

## Author Contributions

ND wrote the article. AB, LB, PM, MM, FM, and UP critically revised the article. All authors contributed to the article and approved the submitted version.

## Conflict of Interest

The authors declare that the research was conducted in the absence of any commercial or financial relationships that could be construed as a potential conflict of interest. The reviewer DD declared a shared affiliation with one of the authors, UP, to the handling editor at time of review.

## Footnotes

^1^CNN. *A Pug in North Carolina May be the First Dog in US to Test Positive for Coronavirus*. Available online at: https://time.com/5828413/dog-coronavirus/ (accessed April 30, 2020).

^2^Confirmation of COVID-19 in Pet Dog in New York USDA Animal and Plant Health Inspection Service sent this bulletin at 06/02/2020 04:10 PM EDT. Available online at: https://content.govdelivery.com/accounts/USDAAPHIS/bulletins/28ead4f (accessed December 14, 2020).

^3^South China Morning Post. *Coronavirus: Pet Cat in Hong Kong Tests Positive After Owner Becomes Infected*. Available online at: https://www.scmp.com/news/hong-kong/health-environment/article/3077802/coronavirus-pet-cat-hong-kong-tests-positive (accessed April 18, 2020).

^4^World Health Organization. *Consensus Document on the Epidemiology of Severe Acute Respiratory Syndrome (SARS)*. Available online at: https://apps.who.int/iris/handle/10665/70863 (accessed April 18, 2020).
